# Haemocompatibility of Modified Nanodiamonds

**DOI:** 10.3390/ma10040352

**Published:** 2017-03-28

**Authors:** Michał Wąsowicz, Mateusz Ficek, Maciej S. Wróbel, Ruchira Chakraborty, Dror Fixler, Paweł Wierzba, Małgorzata Jędrzejewska-Szczerska

**Affiliations:** 1 Department of Morphological Sciences, Faculty of Veterinary Medicine, Warsaw University of Life Sciences, Nowoursynowska 159, 02-776 Warszawa, Poland; michal.wasowicz@interia.eu; 2 Department of Metrology and Optoelectronics, Faculty of Electronics, Telecommunications and Informatics, Gdańsk University of Technology, Narutowicza 11/12, 80-233 Gdańsk, Poland; mateuszficek@gmail.com (M.F.); maciejswrobel@gmail.com (M.S.W.); pwierzba@eti.pg.gda.pl (P.W.); 3 Faculty of Engineering and Institute of Nanotechnology and Advanced Materials, Bar-Ilan University, Ramat Gan 5290002, Israel; ruchira1101@gmail.com (R.C.); Dror.Fixler@biu.ac.il (D.F.)

**Keywords:** haemocompatibility, nanodiamonds, spectroscopy, blood, biophotonics

## Abstract

This study reports the interactions of modified nanodiamond particles in vitro with human blood. Modifications performed on the nanodiamond particles include oxygenation with a chemical method and hydrogenation upon chemical vapor deposition (CVD) plasma treatment. Such nanodiamonds were later incubated in whole human blood for different time intervals, ranging from 5 min to 5 h. The morphology of red blood cells was assessed along with spectral measurements and determination of haemolysis. The results showed that no more than 3% of cells were affected by the nanodiamonds. Specific modifications of the nanodiamonds give us the possibility to obtain nanoparticles which are biocompatible with human blood. They can form a basis for the development of nanoscale biomarkers and parts of sensing systems and devices useful in biomedical environments.

## 1. Introduction

A rapid growth of biomedical research involves the development of imaging and sensing platforms, such as nanoparticles, for biomedical applications [1,2,3]. One such type of nanoparticles are nanodiamonds, which are an allotropic form of carbon whose core consists of the sp^3^ orbital crystal structure and the sp^2^ phase forms most of their surface. The interest in this particular type of nanoparticles comes from its unique characteristics, such as hardness, fine-tuning of their size distributions, emission of strong fluorescence, color centers such as nitrogen vacancy (NV)-centers, photostability [4,5], etc. Moreover, nanodiamonds (NDs) in comparison with fluorescent nanodyes and nanoprobes are more inert and their properties are not changed by external proteins [1]. The compatibility of blood is an important factor regarding the use of materials in blood-related applications, such as biosensing, drug delivery [6], the use of biomarkers for imaging [7,8,9], and other bioapplications [10,11]. It involves several complicated interactions between their surfaces with cellular and other components of blood [12,13,14]. With the increasing push towards the development of novel and more advanced carbon-based biosensing or medicinal products, the issue of biocompatibility has been receiving more attention [15,16]. Many research groups have been dealing with biocompatibility issues involving different biological objects, such as cells, blood, and microorganisms [17,18,19,20]. While the majority of studies present a positive opinion on nanodiamond compatibility in general [21,22,23], this is not always the case. Some studies differ in their opinions, showing negative side effects of nanodiamonds [24,25,26,27]. Thus, we investigate the biocompatibility of such nanodiamonds, while also taking into account the different possible modifications of nanodiamonds, including changing their surface chemistry to a hydrogenated or oxygenated state. We have investigated the in vitro interactions of nanodiamonds with human blood, without any sort of blood processing such as dilution or washing erythrocytes [28,29].

## 2. Results

First, the selected properties of the developed modified nanodiamond suspensions, as described in the experimental part of this study, are presented. Then we present the interactions of the nanodiamonds with human blood, the optical inspection of blood slides, absorbance, and haemolysis studies.

### 2.1. Properties of Nanodiamond Suspensions

**Figure 1:**
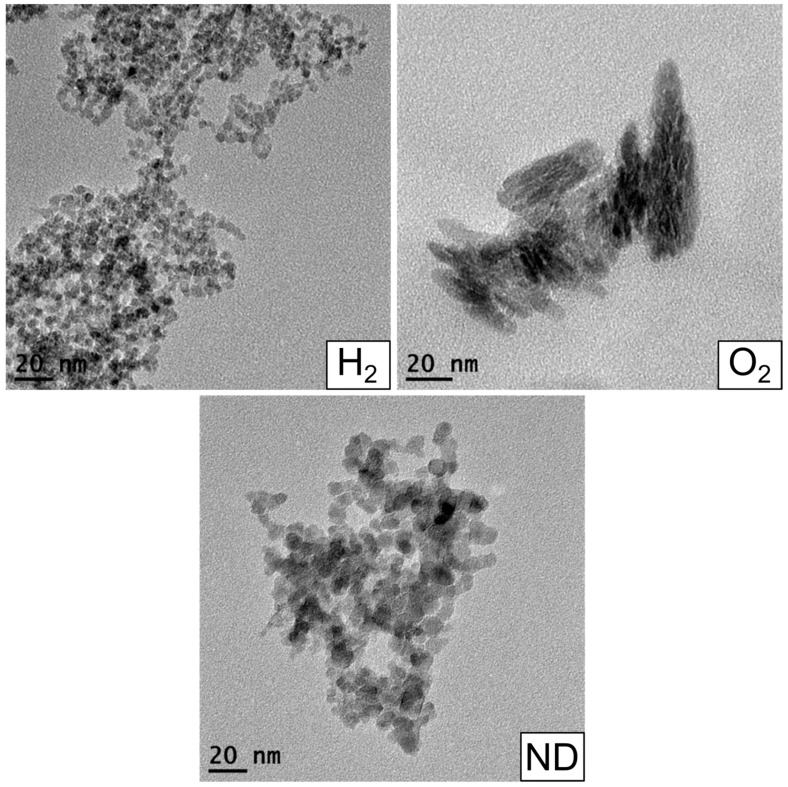
TEM images of oxygenated nanodiamond particles (O_2_), hydrogenated nanodiamond particles (H_2_), and unmodified nanodiamonds (ND).

**Figure 2:**
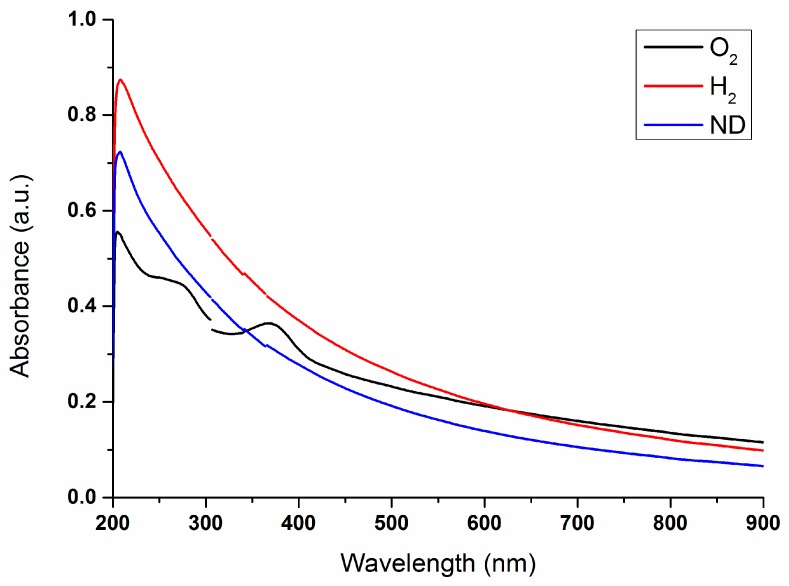
Absorbance spectra of oxygenated nanodiamond particles (ND 1), hydrogenated nanodiamond particles (ND 2), and unmodified nanodiamonds (ND 3).

**Figure 3:**
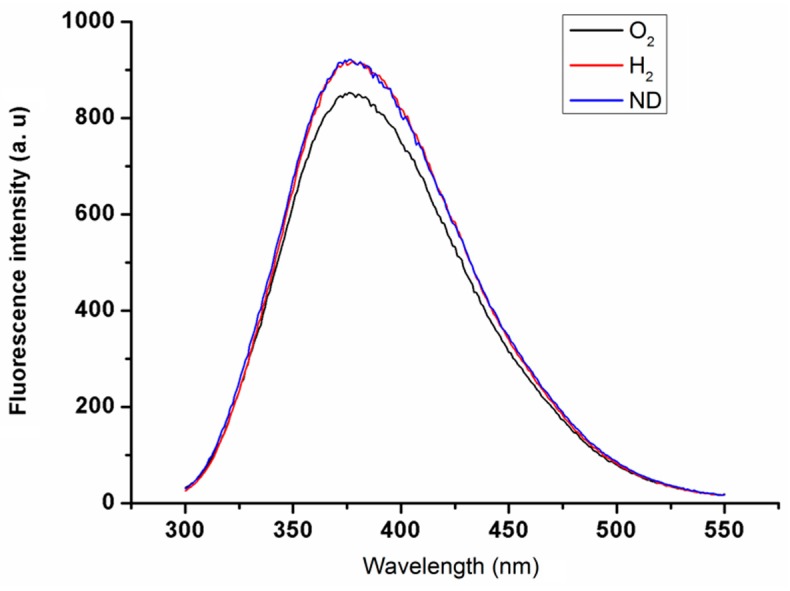
Fluorescence spectra of oxygenated nanodiamond particles (O_2_), hydrogenated nanodiamond particles (H_2_), and unmodified nanodiamonds (ND).

Characterization of the nanodiamond materials was performed in three steps. First, TEM imaging (Figure 1) was used to determine the size and shape of the nanodiamond particles. Second, absorbance spectroscopy (Figure 2) was used to confirm that the modification of the nanodiamond surface yielded the expected results. Third, the fluorescence (Figure 3) of oxygenated (O_2_), hydrogenated (H_2_), and unmodified nanodiamonds (ND) was investigated.

First, the size of the particles were determined through TEM (JEOL JEM-1400) studies. For this study, 10 μL of all the samples were vacuum dried on carbon coated copper grids. Representative images of all three nanodiamond types are presented in Figure 1. The TEM image of H_2_ showed that the size of the particles was around 5–6 nm and that the particles are mainly spherical in shape. In the case of ND 2, the size of the nanoparticle agglomerates were found to be around 10–15 nm. ND 3 particles were also found to be of 10 nm size and they are also formed spherical shaped agglomerates.

Second, 1 mL of each sample was taken for absorbance study through a Shimadzu spectrophotometer (UV-1601). The resulting absorbance spectra are presented in Figure 2. The discontinuities at 305 nm and 365 nm are due to the spectrometer lamp/detector change which was partly corrected. 

For all three investigated nanoparticles, a steady increase in absorbance can be seen, increasing with shorter wavelengths. This is a typical and expected effect arising due to elastic scattering, especially evident at the shortest wavelengths due to strong Rayleigh scattering dependence on the wavelength. Here, hydrogenated nanodiamond particles (H_2_) had the highest absorption, followed by unmodified nanodiamonds (ND) and oxygenated nanodiamond particles (O_2_).

At wavelengths of 268 nm and 360 nm, the oxygenated nanodiamond particles had two absorption peaks which were not observed in the other two. This can be most likely attributed to acid remnants from the chemical oxidation process.

Third, 1 mL of each sample was taken for study by a Varian Cary Eclipse Fluorimeter. The resulting fluorescence spectra are presented in Figure 3. The excitation maxima were observed around 284.5 nm for O_2_ and around 282.4 and 282.8 nm respectively for H_2_ and ND.

All three nanodiamonds had fluorescence emission maxima at 376 nm. The unmodified nanodiamond (ND) and hydrogenated nanodiamond (H_2_) had almost the same spectral values even at the maxima. The oxygenated nanodiamond (O_2_) showed a slightly lower fluorescence intensity than the other two at the maxima.

### 2.2. Interactions of Nanodiamonds with Human Blood

**Figure 4:**
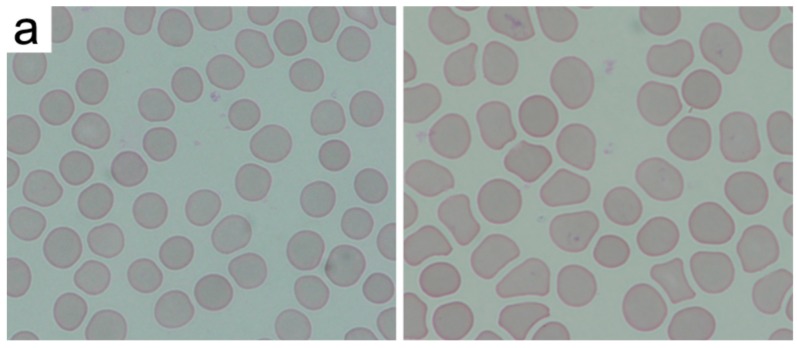

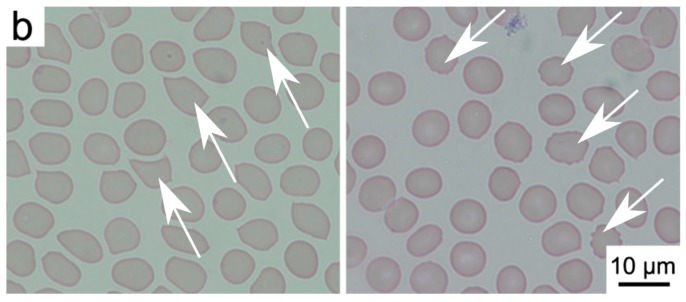
Examples of normal and abnormal erythrocytes: (**a**) normal erythrocytes with typical mechanical deformations; (**b**) echinocytes with deformed membranes (indicated by arrows).

We have investigated the changes induced in the morphology of red blood cells through visual inspection by a clinician. After the staining procedure, the samples were analyzed under a bright field optical microscope. Figure 4 and the supplementary images (Supplementary Information, Figures S1–S31) present the representative microscopic images after the inspection of a human blood film. The differential analysis of red blood cells was performed over the blood smear area in order to detect the atypical cells that changed due to the introduction of NDs. Examples of normal and abnormal erythrocytes are presented in Figure 4. Normal red blood cells are shown with mechanical deformations which normally occur during the blood smear and May-Grünwald and Giemsa (*MGG*) staining procedure, as well as the examples of echinocytes. We have conducted a short, as well as a long-time incubation, to assess the rapid and long-term effects of nanodiamonds on erythrocytes. Mostly normal erythrocytes were found in the blood smears—oval cells with similar diameters and shapes. The brighter areas in the center of the cell are the indicator of normocytes. Abnormal cells were exclusively detected in the form of echinocytes. The correct, round shape of the erythrocytes may undergo atrophy, causing the emergence of numerous extensions of the cell membrane. Changes of the cell membranes of erythrocytes could be related to the effects of nanodiamonds in vitro.

**Figure 5:**
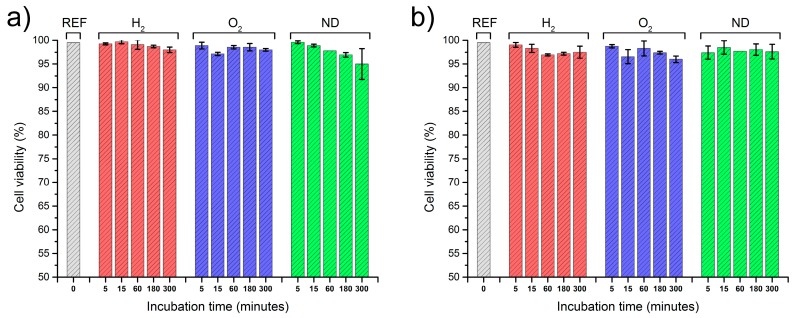
Cumulative statistics on cell viability after interaction with nanodiamonds (*n* = 10). The (**a**) plot presents a concentration of 38 ng/μL; and (**b**) 167 ng/μL. Data is presented as mean ± standard deviation. The results of one-way ANOVA yielded the differences as not statistically significant at the *p* < 0.05 confidence level.

**Figure 6:**
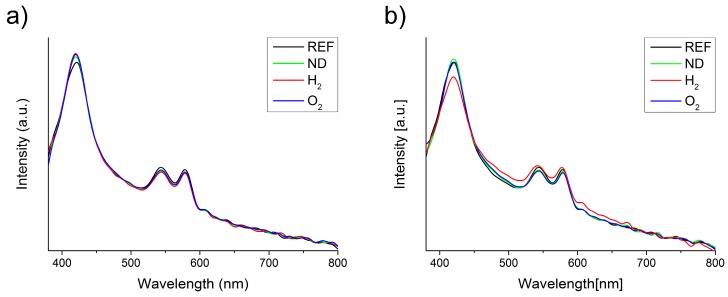
Absorption spectra of investigated blood samples with concentrations of (**a**) 38 ng/μL; and (**b**) 167 ng/μL; Blood with NDs: unmodified (ND), hydrogen-terminated (H_2_), and oxygen-terminated (O_2_), as well as the reference blood sample without NDs (REF).

**Figure 7:**
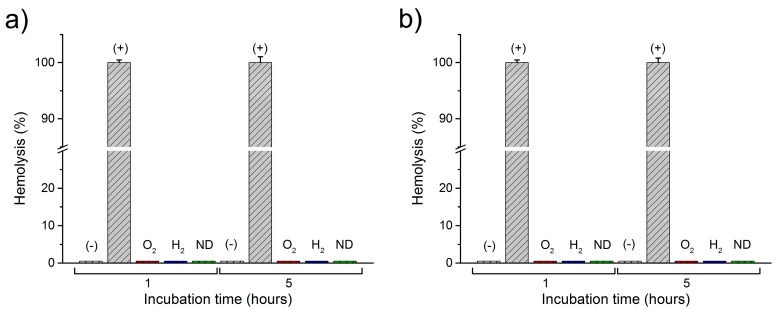
Hemolysis assessment of investigated blood samples with concentrations of (**a**) 38 ng/μL; and (**b**) 167 ng/μL; Blood with NDs: unmodified (ND), hydrogen-terminated (H_2_), and oxygen-terminated (O_2_), as well as the reference blood sample without NDs, negative control (−) with saline, and positive control (+) with Triton X-100.

A statistical analysis using one-way ANOVA for the number of abnormal cells is presented in Figure 5 for the reference blood sample (REF), as well as for the modified nanodiamonds: hydrogenated (H_2_) and oxygenated (O_2_). The data is presented as the mean and error bars correspond to ±standard deviation (SD), yielding variation between patients not more than roughly 3% (as shown in Figure 5). A similar level of abnormal cells is evident in both the reference sample as well as the modified nanodiamonds. The number of observed echinocytes was very low which was not considered as statistically significant quantities at a level of confidence of *p* < 0.05. Changes of the concentration of nanodiamonds also did not cause any statistically significant number of affected cells at the *p* < 0.05 confidence level. In general, the morphology of the red blood cells remained unchanged after the introduction of all nanodiamond types, concentrations, and incubation times. Figure 6 presents the absorption spectra of samples with two concentrations of unmodified nanodiamonds (ND), as well as hydrogen-terminated (H_2_), and oxygen-terminated (O_2_) nanodiamonds. The spectra show minimal differences between the reference sample and each investigated concentration and incubation time. Hemoglobin retained its oxygenated state which is indicated by the characteristic two absorption bands at about 540 nm and 570 nm. Therefore, we conclude that no change in the hemoglobin conformation state occurred due to the introduction of nanodiamonds with different modifications to the whole human blood. Figure 7 presents the results of the hemolysis assessment with the standard reference method through the reduction of hemoglobin to azidemethemoglobin and subsequent spectrophotometric measurement, which yielded 0% of hemolysis upon the introduction of NDs in a short (1 h) as well as long-term (5 h) incubation period. Thus, we can conclude that the process of nanodiamond modification, including their purification and change of the nanodiamond surface chemistry, yielded the hemocompatible nanoparticles.

## 3. Discussion

The viability of nanodiamonds for a multitude of applications such as drug delivery, optical imaging, and sensing makes them a very promising material for biomedical applications. Yet there is a dispute over the actual biocompatibility of nanodiamonds interacting with different biological objects, such as cancerous and normal cells, blood cells, microorganisms, etc. We have therefore conducted an investigation into this issue, focusing especially on the case of nanodiamond compatibility with red blood cells. We used commercial detonation nanodiamonds as a reference and modified their surface using chemical and plasma methods which yielded H_2_ and O_2_ termination of the nanodiamond surfaces. Furthermore, we have conducted the assessment of their biocompatibility with different ND concentrations, as well as with different time periods. The observed cell viability data were not statistically significant at the *p* < 0.05 confidence level, thus there is no adverse effect of ND interactions with whole blood at the chosen concentrations. The short- and long-time investigations showed that modification of the nanodiamonds give us the possibility to obtain nanoparticles which are biocompatible with human blood (haemocompatibility). Therefore, we can use them as a basis for designing new biomarkers dedicated to bioimaging. Furthermore, we perceive their eventual usefulness in biomedical instrumentation as photonic sensors [30,31].

## 4. Materials and Methods

### 4.1. Materials

Nanodiamond particles were produced from carbon in high-energy explosives [32,33]. In our experiments, commercial nanodiamond powder (Detonation Nanodiamond Powder, Adamas Nanotechnology, Raleigh, NC, USA) was used. The characteristic size of the primary particles of detonation nanodiamond (DND) was from 4 to 5 nm, however they formed tight aggregates during synthesis and purification [34]. The raw DND powder (not fractionated and not specifically functionalized) was available both with positive zeta potential and negative zeta potential (positive and negative surface electrostatic charge for the particles dispersed in deionized water, correspondingly). Those nanodiamond particles were used for reference samples and for future surface modification. We have developed two types of nanodiamonds with different surface terminations: H_2_ and O_2_. Chemical and plasma modification techniques were used to obtain such surface terminations of the nanodiamond powders. The different surface terminations changed the sign of the zeta potential. The sign of the zeta potential depends on the type of surface groups (basic or acidic) and is important for many applications (adsorption of biomolecules, electrophoretic behavior, electroplating, etc.) [35].

Chemical pre-treatment of the nanodiamond particles was applied to obtain an oxygen-terminated surface and to etch the sp^2^ phase impurities. The organic impurities were dissolved in hot “piranha” solution (H_2_O_2_:H_2_SO_4_/1:3) at 200 °C. The process continued for about 60 min, and then the sample was rinsed with water and methanol, and dried [36].

Microwave hydrogen plasma treatment was performed using a microwave plasma-assisted chemical vapor deposition (MW PACVD) system (Seki Technotron AX5400S, Tokyo, Japan). The highly excited plasma was generated with microwave radiation (2.45 GHz) and an optimized power of 1300 W. During the process, the molybdenum stage was heated up to 500 °C by an induction heater and was controlled by the thermocouple. The total hydrogen flow was kept at 300 sccm. The hydrogenation time was kept at 10 min. Thus, the resulting nanodiamond surface was predominantly hydrogen-terminated [37]. After the growth process, the substrate temperature was slowly reduced (5 °C/min) down to room temperature. The temperature was adjusted by the simultaneous lowering of the microwave power and current of the induction heater. 

Aforementioned procedures yield the modification of the nanodiamond surface as oxygenated and hydrogenated. It has been shown that similar methods yield such modifications, resulting in the surface of oxygenated nanodiamonds becoming hydrophilic, while the surface of the hydrogenated nanodiamonds becomes hydrophobic [36,37,38,39].

### 4.2. Methods

Two modes of the hemocompatibility assessment were utilized: (1) a bright-field light microscope analysis of peripheral blood smears with May-Grünwald and Giemsa (MGG) staining, morphologically analyzed by the clinician; (2) absorption spectroscopy of samples in a visible wavelength range and spectrophotometric hemolysis assessment.

We have investigated the changes induced in the morphology of red blood cells through visual inspection by a trained clinician (see the Acknowledgements section). The laboratory experiments were performed according to well established (gold standard) methods [40,41,42,43,44,45,46]. We have prepared at least two blood smears for each of the analyzed samples. The smears were stained following the MGG protocol. After the staining procedure, the samples were analyzed under a bright field optical microscope (Nikon Eclipse 80, magnification: 1000× with immersion oil, refractive index *n* = 1.518). The differential analysis of red blood cells was performed over the blood smear area in order to detect the atypical cells changed due to the ND introduction. The counting area was considered as laying between the thick part of the slides, where the cells are overlaid upon each other making the observations of the individual cells impossible, and the feathered edge where the leukocytes undergo mechanical deformation and the parlor of erythrocytes cannot be evaluated. In routine laboratory practice, a number of abnormal cells is evaluated per 100 individual cells in the observed area. However, to improve the statistical significance of the results, we have investigated per 1000 cells in the observable area, thus increasing the amount of cells counted by an order of magnitude. Three areas were chosen at random over the smear area, and the cumulative results were averaged. Likewise, the second slide was inspected, and the results were averaged. The error bars in Figure 5 correspond to the variability between other patients’ blood following the same inspection methodology. The investigation was focused on morphological features of the erythrocytes, such as their size, shape, and chromaticity. The most notable result was the presence of echinocytes with visible spike-like features present on the erythrocyte membrane, as seen in Figure 4b. We have conducted a short as well as a long-time study, to assess the rapid and long-term effects of nanodiamonds.

The absorption spectroscopy was used to measure the absorbance (or optical density) of the blood sample, which indicates the conformation state of the hemoglobin. Absorption spectra were recorded for samples of the human blood and blood with ND particles, to assess the induced changes. For this purpose, a custom-built spectrometer set-up was used, as described in detail in our previous publications [47]. The set-up consisted of a compact spectrometer (Flame, Ocean Optics Inc., Dunedin, FL, USA) with a 190–1100 nm range, tungsten-halogen light source (BP101, B&W Tek, Newark, DE, USA), optical fibers, and a sample holder. To avoid the introduction of any sort of material, including dilution, the blood with nanodiamonds could not be measured in a regular spectrometer cuvette, due to the huge absorption at long optical paths in the cuvette. The samples were smeared on a microscopic slide and covered with a cover glass. A vast number of spectra were collected with 10 ms of integration time and 10 accumulations each. Such an approach was necessary to avoid the sample dilution. Furthermore, the spectra were averaged over the area to ensure uniformity of the sample and optical path length. The dataset was later treated with a sample normal variate (SNV) normalization to reduce the variations in optical path length as well as the effects of scattering. The spectra were smoothed with the Savitzky-Golay algorithm, and baseline-subtracted.

Hemolysis assessment of blood interacting with NDs was performed following standard procedures [48,49,50]. Two additional samples were prepared: positive (+) and negative (−) control. In the negative hemolysis control, the blood was mixed with saline solution to contain the erythrocytes intact and was assumed as 0% hemolysis. In the positive hemolysis control, blood was mixed with Triton X-100 (AppliChem, GmbH, DE) at 1% (*v/v*) concentration, to rupture the cell walls and release free hemoglobin, assuming 100% hemolysis. Samples of human blood with introduced to three types of nanodiamonds and control samples were incubated for a specific period of time (1 h and 5 h) in standard conditions, and later centrifuged at 4000 rpm. The supernatant plasma was taken for measurements of the free hemoglobin concentration, which is the sign of hemolysis. Hemoglobin concentration was measured using a standard method based on spectrophotometric measurement of azidemethemoglobin using a certified hemoglobinometer (HemoCue AB, SWE, Ängelholm, Sweden). Free hemoglobin is converted from the ferrous to ferric state by sodium nitride forming methemoglobin, which in turn forms azidemethemoglobin by the combination with azide [51,52].

### 4.3. Experimental

The whole blood samples of venous blood used in this study were collected up to 6 h prior to the beginning of the experiments. The samples were collected into 2 mL vials with EDTA as an anticoagulant, from healthy volunteers following the standard procedures. A peripheral blood stain was conducted on whole human blood as a reference sample (REF). Next, the suspended nanodiamonds (1000 ng/μL) were introduced into 500 μL aliquots of the blood samples. The volume of the suspension was 20 μL and 100 μL, resulting in concentrations of 38 ng/μL and 167 ng/μL in the samples. Three types of nanodiamonds were tested overall, the unmodified stock nanodiamonds (ND), and the two types with modified surface chemistry: hydrogen-terminated (H_2_) and oxygen-terminated (O_2_) nanodiamonds. The samples were left to incubate for a specified time (from 5 min to 5 h), and after each time point, the peripheral blood stain was performed. This allowed for a short- as well as a long-term evaluation of nanodiamond interactions with red blood cells. The stains were performed after the incubation continued for the chosen time points: 5 min, 15 min, 60 min, 180 min, and 300 min. To ensure the reliability and validity of the study, at least 10 blood stains were always performed for each sample from at least 10 volunteers. The blood smears were stained according to the May-Grünwald and Giemsa (MGG) staining procedure. The samples were checked for hemolysis after 1 h and 5 h of incubation, and their absorption spectra were collected. The interaction time between the cells and the nanodiamond in in vivo conditions can be much longer than the time limit of 300 min selected for this study. In principle, it is possible that with longer interaction time that some effect (or stronger effect) could be observed.

## 5. Conclusions

We have assessed the haemocompatibility of modified NDs with respect to the red blood cells. Three types of NDs were investigated: the stock unmodified NDs and two modifications to their surface chemistry: plasma hydrogenation and chemical oxygenation were performed to create oxygen and hydrogen-terminated surfaces of NDs. The interactions of NDswith erythrocytes was assessed through optical investigation by the clinician, as well as haemolysis assay. The numbers of affected erythrocytes in all cases were very low, which was not considered by a one-way ANOVA as statistically significant differences at significance level of *p* < 0.05. The haemolysis did not occur. Therefore, all three types of NDs were considered haemocompatible, which permits their contact with blood.

## Supplementary Materials

The following are available online at www.mdpi.com/1996-1944/10/4/352/s1. Table S1: Figure number for each type, Figure S1: Microscopic image of a reference blood sample, Figure S2: Nanodiamond type—ND, concentration—38 ng/µL, incubation time—5 min, Figure S3: Nanodiamond type—ND, concentration—38 ng/µL, incubation time—15 min, Figure S4: Nanodiamond type—ND, concentration—38 ng/µL, incubation time—1 h, Figure S5: Nanodiamond type—ND, concentration—38 ng/µL, incubation time—3 h, Figure S6: Nanodiamond type—ND, concentration—38 ng/µL, incubation time—5 h, Figure S7: Nanodiamond type—O_2_, concentration—38 ng/µL, incubation time—5 min, Figure S8: Nanodiamond type—O_2_, concentration—38 ng/µL, incubation time—15 min, Figure S9: Nanodiamond type—O_2_, concentration—38 ng/µL, incubation time—1 h, Figure S10: Nanodiamond type—O_2_, concentration—38 ng/µL, incubation time—3 h, Figure S11: Nanodiamond type—O_2_, concentration—38 ng/µL, incubation time—5 h, Figure S12: Nanodiamond type—H_2_, concentration—38 ng/µL, incubation time—5 min, Figure S13: Nanodiamond type—H_2_, concentration—38 ng/µL, incubation time—15 min, Figure S14: Nanodiamond type—H_2_, concentration—38 ng/µL, incubation time—1 h, Figure S15: Nanodiamond type—H_2_, concentration—38 ng/µL, incubation time—3 h, Figure S16: Nanodiamond type—H_2_, concentration—38 ng/µL, incubation time—5 h, Figure S17: Nanodiamond type—ND, concentration—167 ng/µL, incubation time—5 min, Figure S18: Nanodiamond type—ND, concentration—167 ng/µL, incubation time—15 min, Figure S19: Nanodiamond type—ND, concentration—167 ng/µL, incubation time—1 h, Figure S20: Nanodiamond type—ND, concentration—167 ng/µL, incubation time—3 h, Figure S21: Nanodiamond type—ND, concentration—167 ng/µL, incubation time—5 h, Figure S22: Nanodiamond type—O_2_, concentration—167 ng/µL, incubation time—5 min, Figure S23: Nanodiamond type—O_2_, concentration—167 ng/µL, incubation time—15 min, Figure S24: Nanodiamond type—O_2_, concentration—167 ng/µL, incubation time—1 h, Figure S25: Nanodiamond type—O_2_, concentration—167 ng/µL, incubation time—3 h, Figure S26: Nanodiamond type—O_2_, concentration—167 ng/µL, incubation time—5 h, Figure S27: Nanodiamond type—H_2_, concentration—167 ng/µL, incubation time—5 min, Figure S28: Nanodiamond type—H_2_, concentration—167 ng/µL, incubation time—15 min, Figure S29: Nanodiamond type—H_2_, concentration—167 ng/µL, incubation time—1 h, Figure S30: Nanodiamond type—H_2_, concentration—167 ng/µL, incubation time—3 h, Figure S31: Nanodiamond type—H_2_, concentration—167 ng/µL, incubation time—5 h.
