# Supplementary material for: Haemocompatibility of Modified Nanodiamonds
Source: Materials (Basel). 2017 Mar 28;10(4):352. doi: 10.3390/ma10040352 (PMC5506996; doi:10.3390/ma10040352)
Supplement: Supplementary file 1 [file materials-10-00352-s001.pdf]

Supplementary Materials: Haemocompatibility of Modified Nanodiamonds

Michał Wąsowicz, Mateusz Ficek, Maciej S. Wróbel, Ruchira Chakraborty, Dror Fixler, Paweł Wierzba and Małgorzata Jędrzejewska-Szczerska

This supplementary material provides high resolution representative microscopic images for a biocompatibility study of the interaction of nanodiamonds with whole human blood. Here, we focus solely on the morphology of RBCs after incubation with different surface terminations of nanoparticles, including ND–stock unmodified nanodiamond particles, O_2–oxygenated nanodiamonds, and H_2–hydrogenated nanodiamonds. The samples were incubated with two concentrations (20 µL and 100 µL) of added nanoparticle suspensions, corresponding to 38 and 167 ng/µL of nanodiamonds. For each series, a reference sample (REF) is provided. The basis for the classification of cells as viable or affected is presented in Figure 1 of the manuscript. Here, Figure S1 provides the reference blood sample, while the Figures S2–S31 provide images for each nanodiamond type, concentration, and incubation time, as described for clarity in Table 1.

Table 1. Figure number for each type.

Nanodiamond Concentration	Nanodiamond Type	Incubation Time				
		5 min	15 min	1 h	2 h	5 h
38 ng/µL	ND	S2	S3	S4	S5	S6
	O_2	S7	S8	S9	S10	S11
	H_2	S12	S13	S14	S15	S16
167 ng/µL	ND	S17	S18	S19	S20	S21
	O_2	S22	S23	S24	S25	S26
	H_2	S27	S28	S29	S30	S31

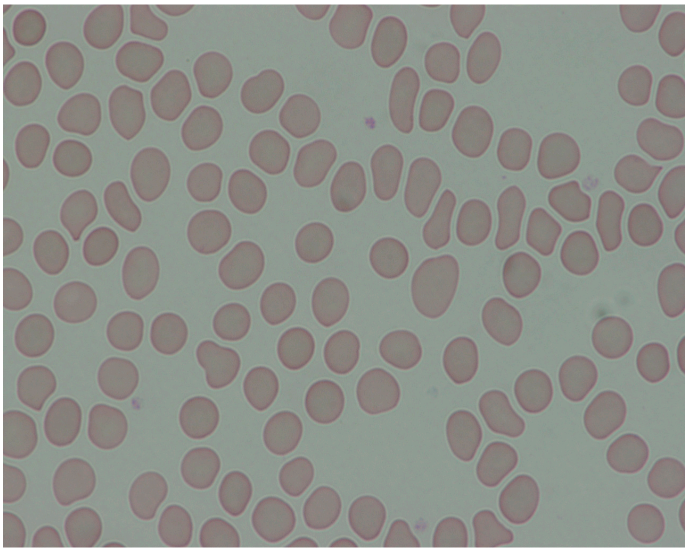

Figure S1. Microscopic image of a reference blood sample.

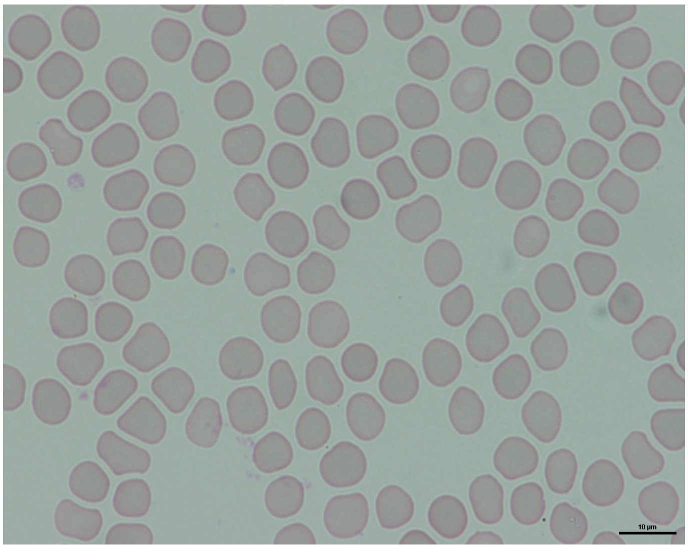

Figure S2. Nanodiamond type—ND, concentration—38 ng/μL, incubation time—5 min.

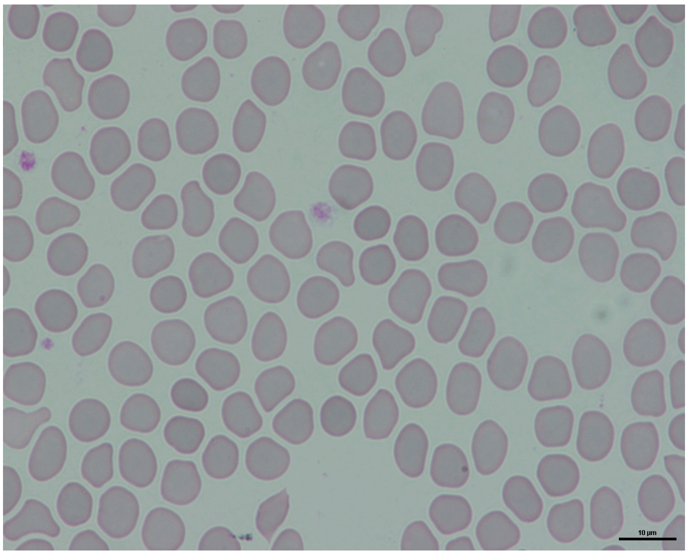

Figure S3. Nanodiamond type—ND, concentration—38 ng/μL, incubation time—15 min.

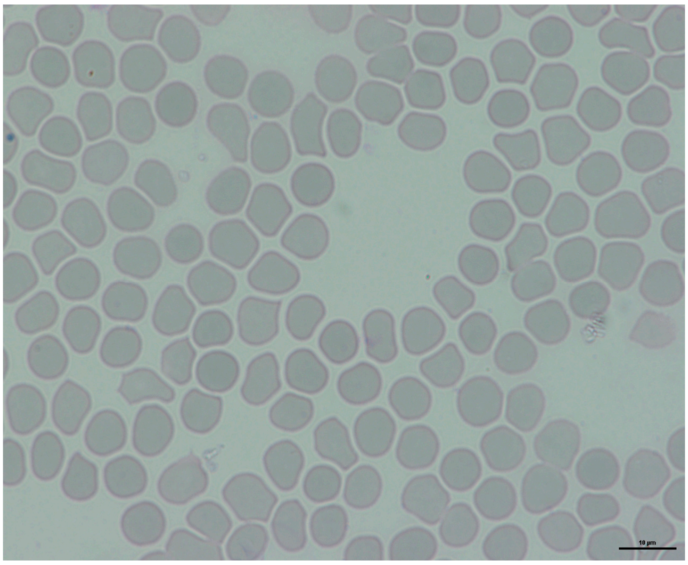

Figure S4. Nanodiamond type—ND, concentration—38 ng/μL, incubation time—1 h.

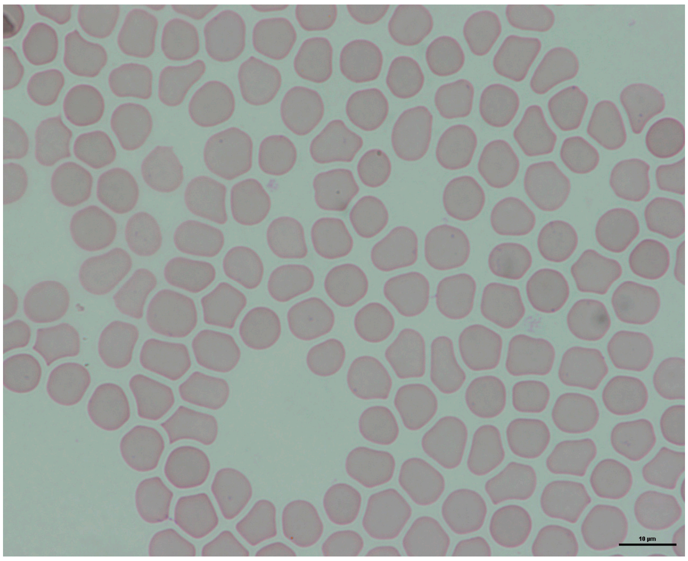

Figure S5. Nanodiamond type—ND, concentration—38 ng/μL, incubation time—3 h.

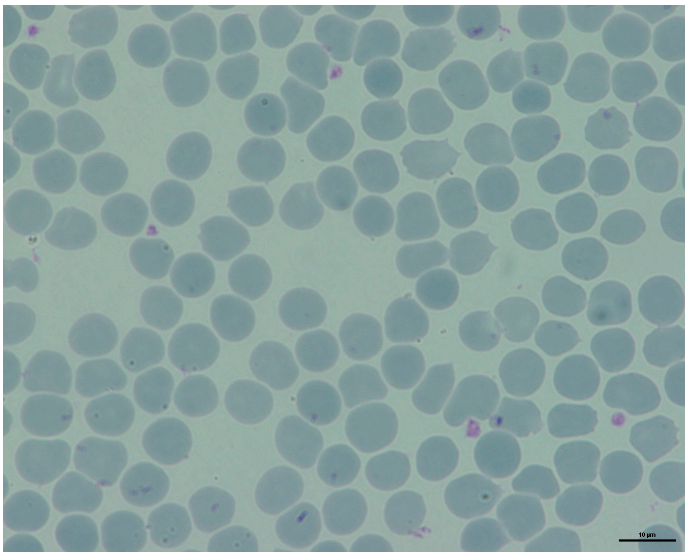

Figure S6. Nanodiamond type—ND, concentration—38 ng/μL, incubation time—5 h.

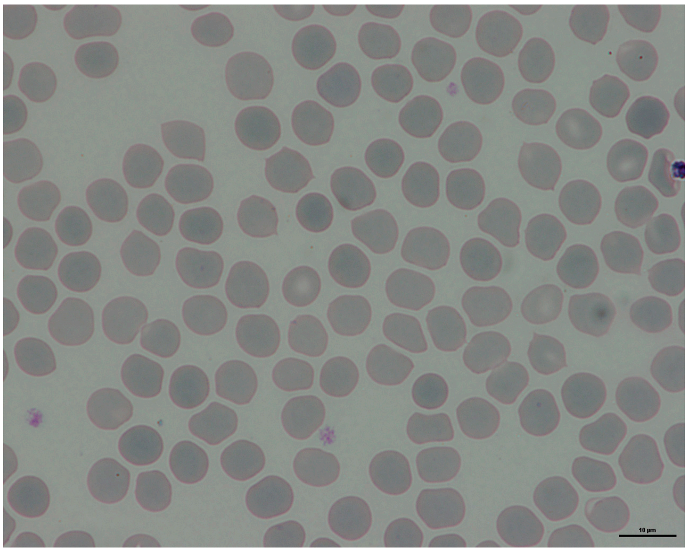

Figure S7. Nanodiamond type—O_2, concentration—38 ng/μL, incubation time—5 min.

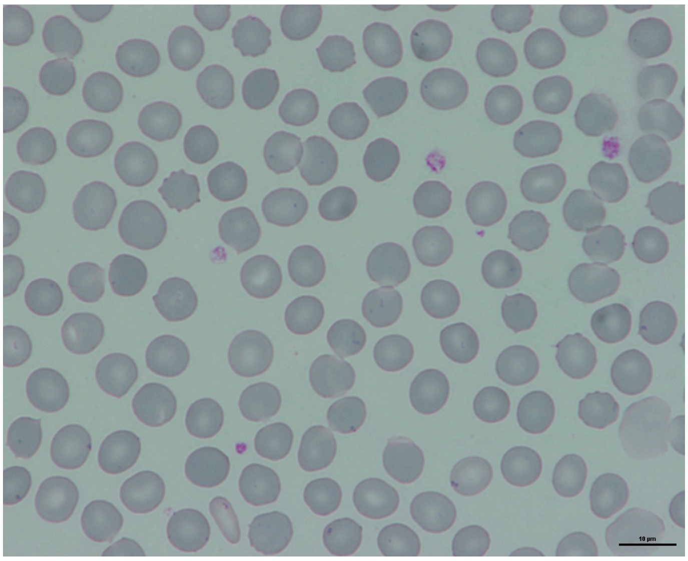

Figure S8. Nanodiamond type—O_2, concentration—38 ng/μL, incubation time—15 min.

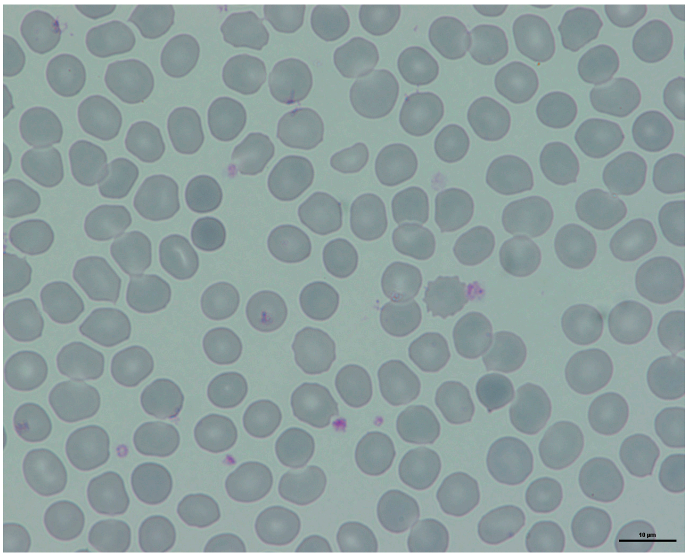

Figure S9. Nanodiamond type—O_2, concentration—38 ng/μL, incubation time—1 h.

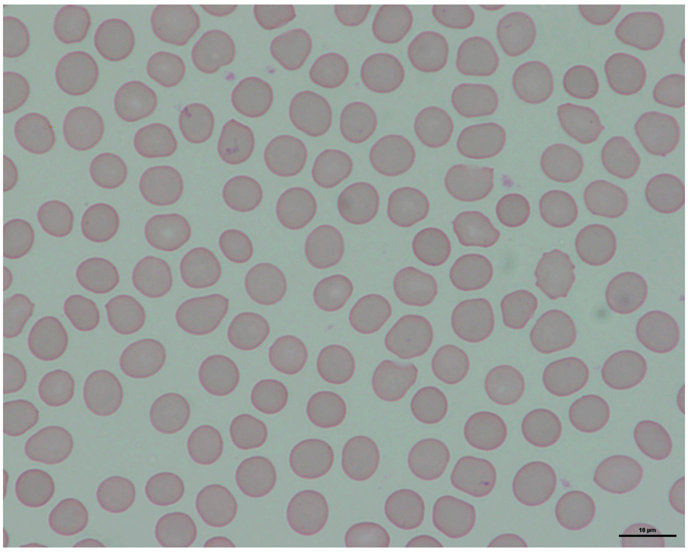

Figure S10. Nanodiamond type—O_2, concentration—38 ng/μL, incubation time—3 h.

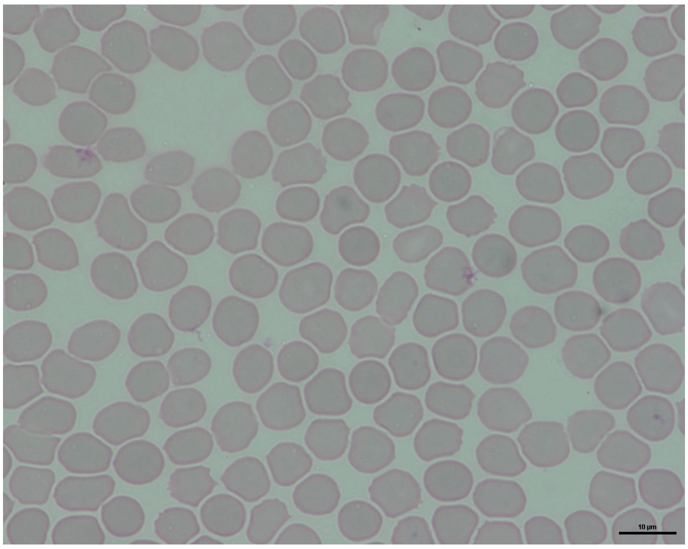

Figure S11. Nanodiamond type—O_2, concentration—38 ng/μL, incubation time—5 h.

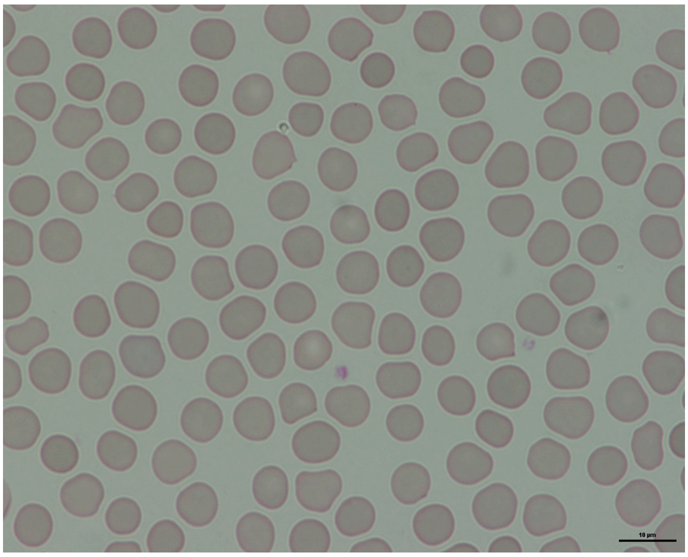

Figure S12. Nanodiamond type—H_2, concentration—38 ng/μL, incubation time—5 min.

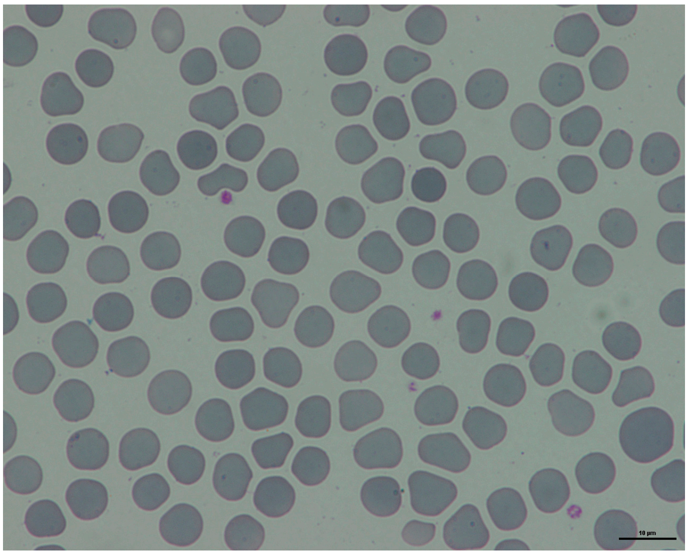

Figure S13. Nanodiamond type—H_2, concentration—38 ng/μL, incubation time—15 min.

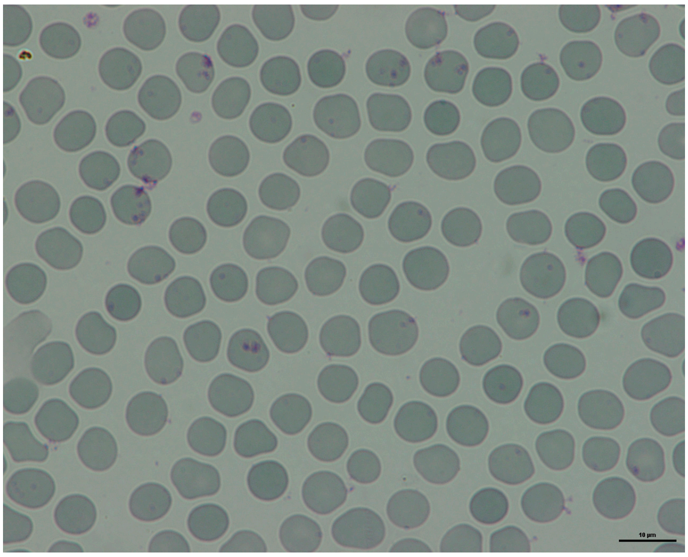

Figure S14. Nanodiamond type—H_2, concentration—38 ng/μL, incubation time—1 h.

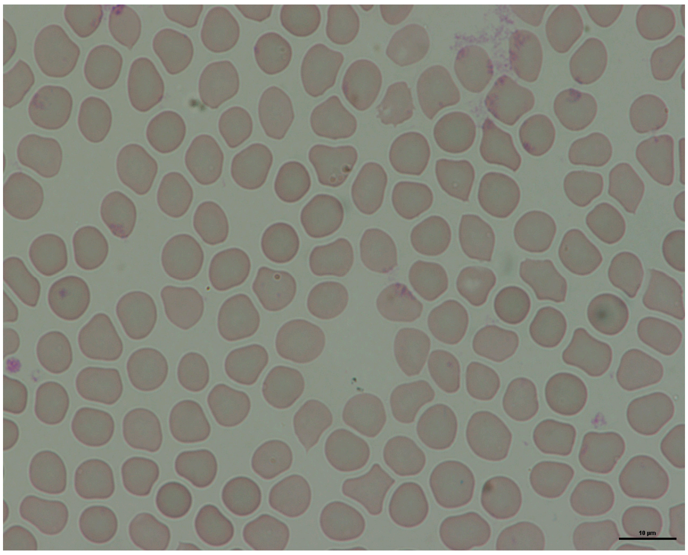

Figure S15. Nanodiamond type—H_2, concentration—38 ng/μL, incubation time—3 h.

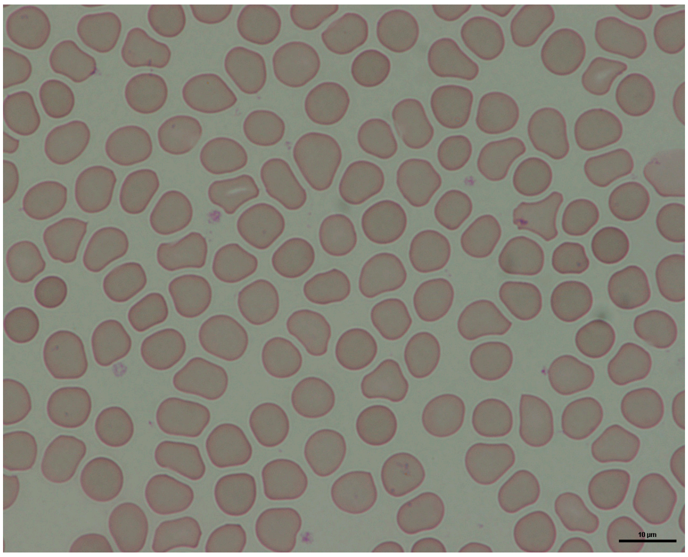

Figure S16. Nanodiamond type—H_2, concentration—38 ng/μL, incubation time—5 h.

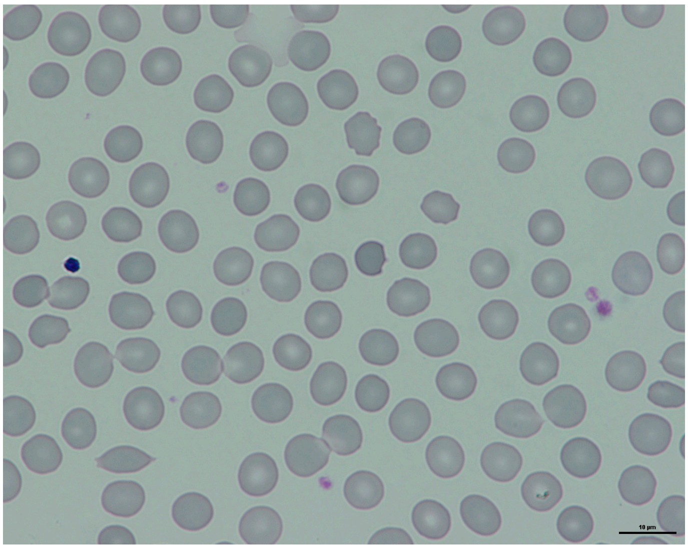

Figure S17. Nanodiamond type—ND, concentration—167 ng/μL, incubation time—5 min.

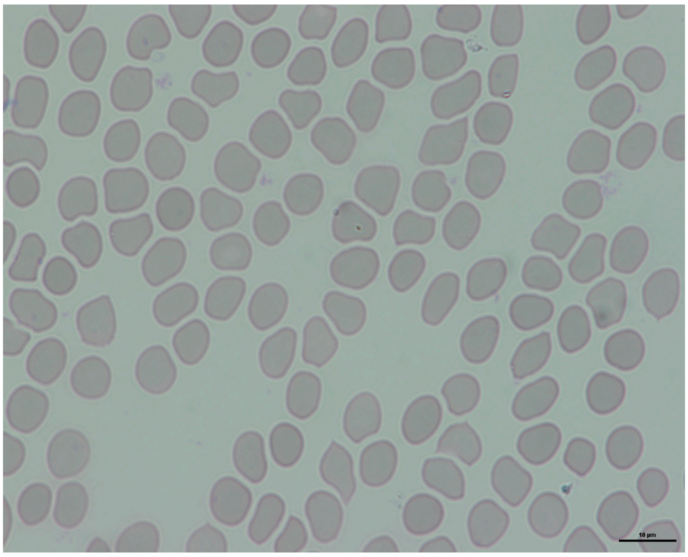

Figure S18. Nanodiamond type—ND, concentration—167 ng/μL, incubation time—15 min.

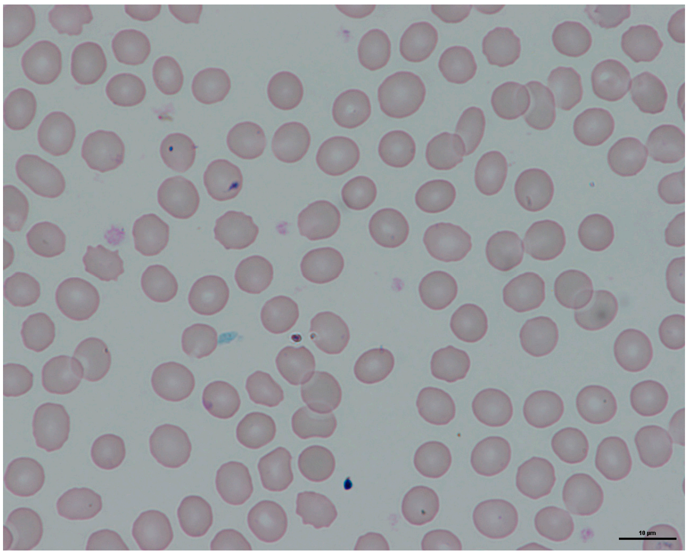

Figure S19. Nanodiamond type—ND, concentration—167 ng/μL, incubation time—1 h.

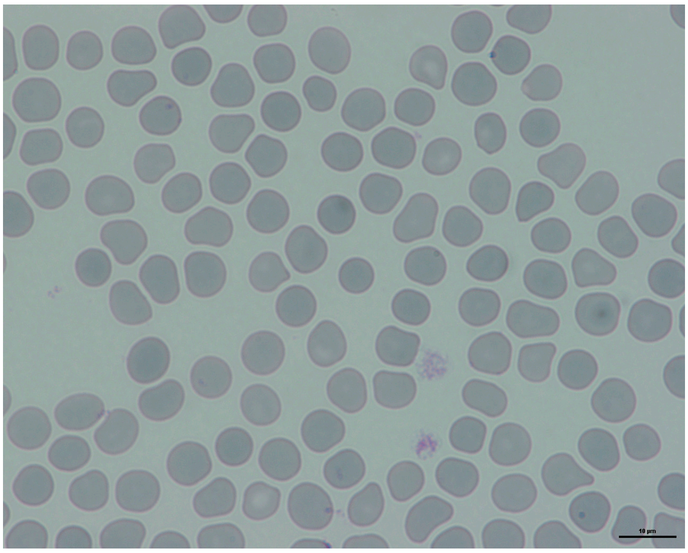

Figure S20. Nanodiamond type—ND, concentration—167 ng/μL, incubation time—3 h.

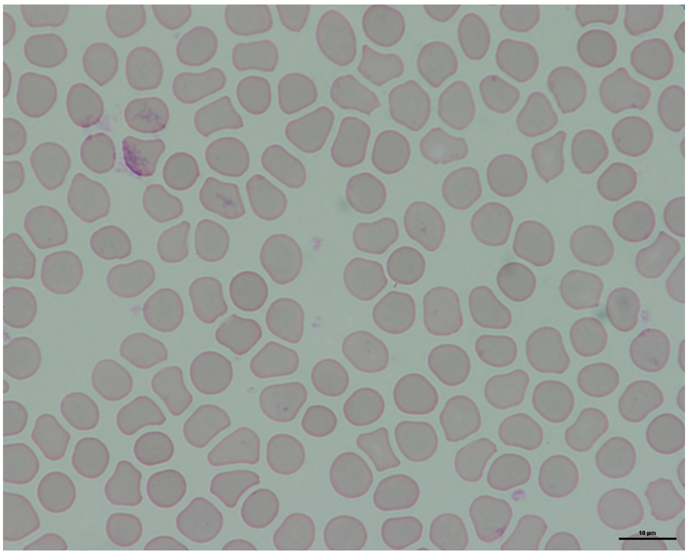

Figure S21. Nanodiamond type—ND, concentration—167 ng/μL, incubation time—5 h.

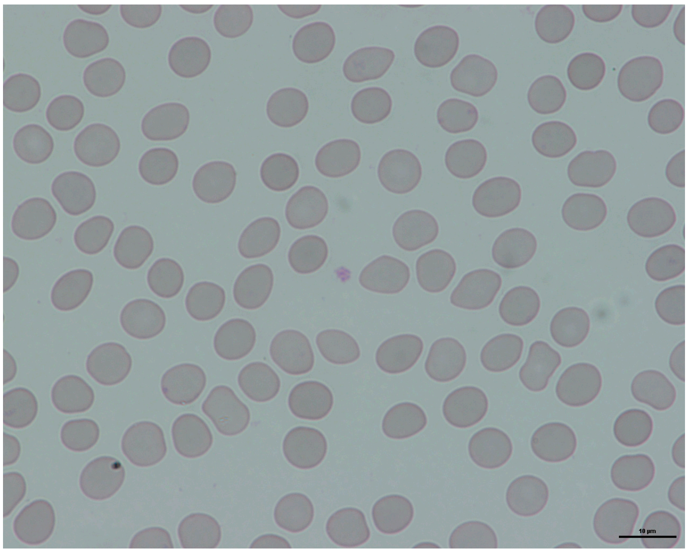

Figure S22. Nanodiamond type—O_2, concentration—167 ng/μL, incubation time—5 min.

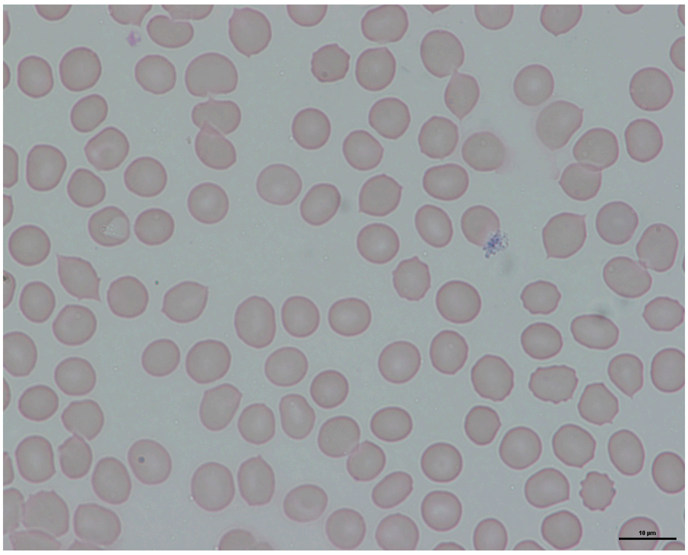

Figure S23. Nanodiamond type—O_2, concentration—167 ng/μL, incubation time—15 min.

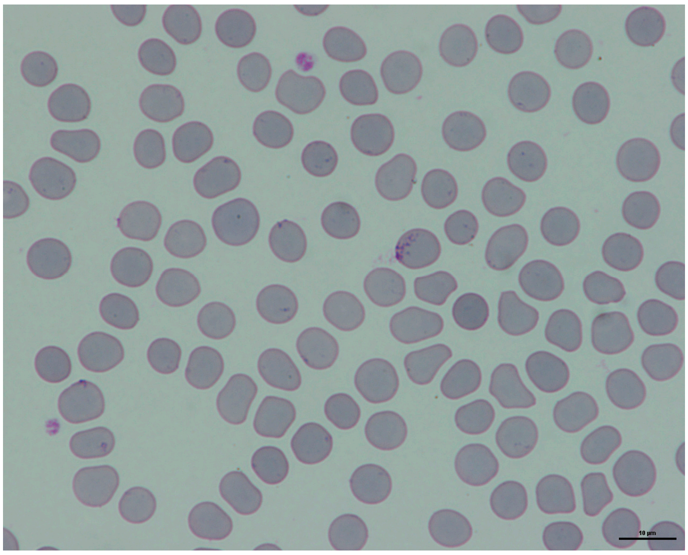

Figure S24. Nanodiamond type—O_2, concentration—167 ng/μL, incubation time—1 h.

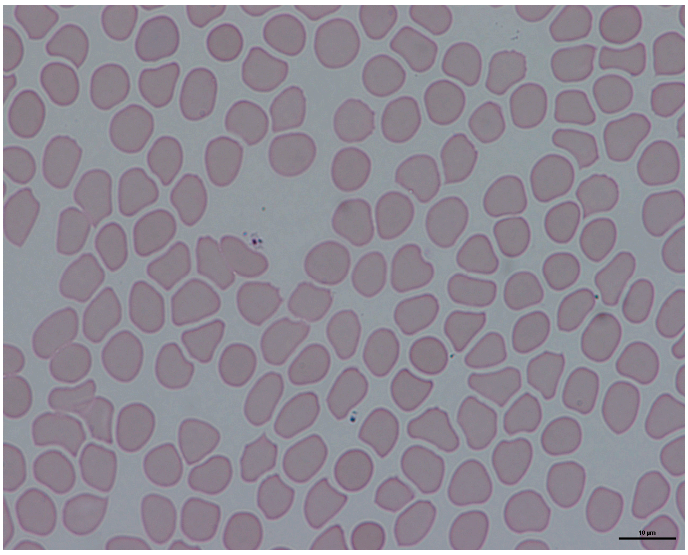

Figure S25. Nanodiamond type—O_2, concentration—167 ng/μL, incubation time—3 h.

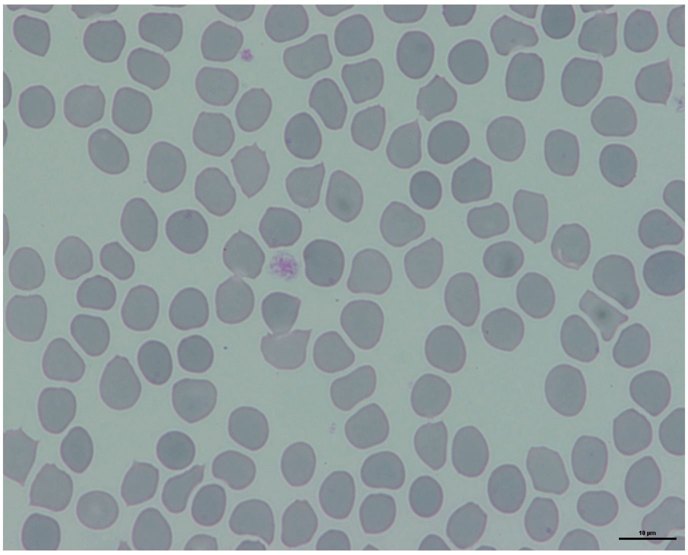

Figure S26. Nanodiamond type—O_2, concentration—167 ng/μL, incubation time—5 h.

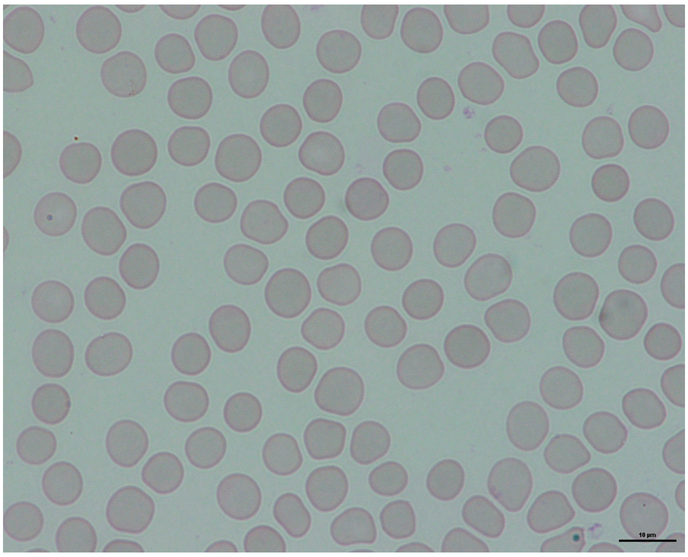

Figure S27. Nanodiamond type—H_2, concentration—167 ng/μL, incubation time—5 min.

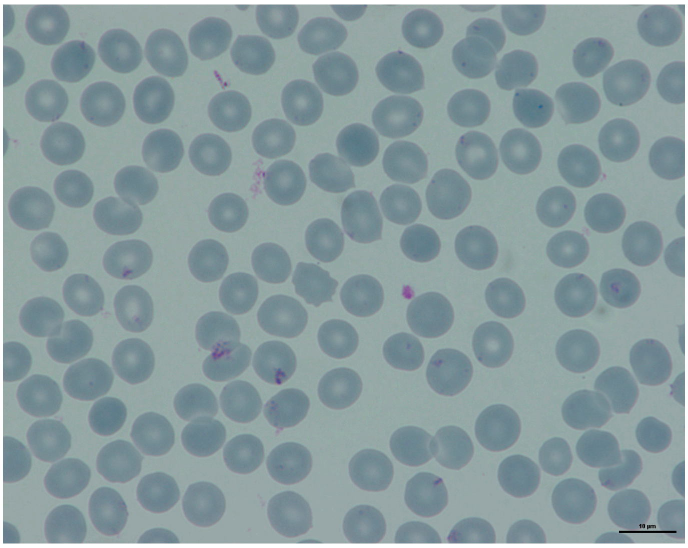

Figure S28. Nanodiamond type—H_2, concentration—167 ng/μL, incubation time—15 min.

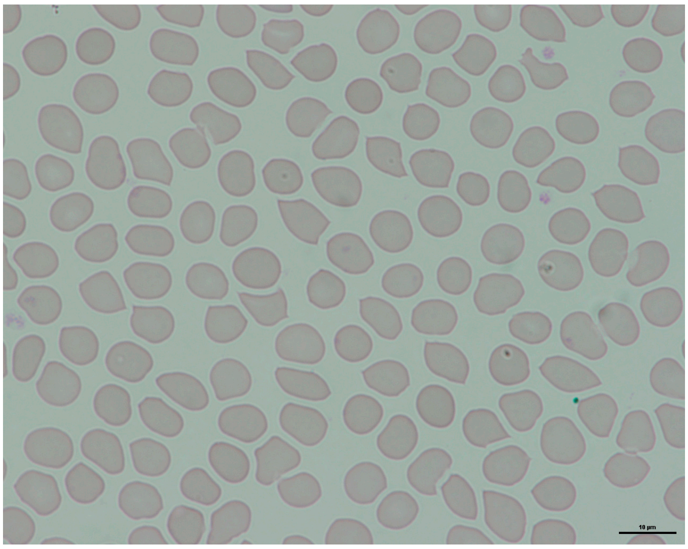

Figure S29. Nanodiamond type—H_2, concentration—167 ng/μL, incubation time—1 h.

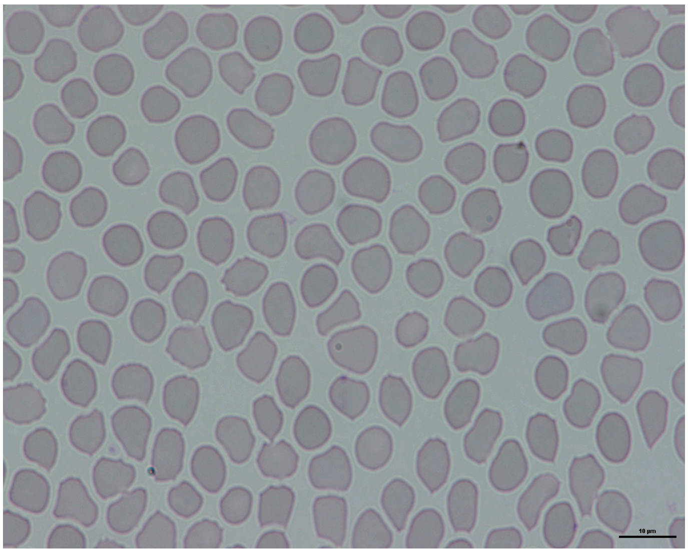

Figure S30. Nanodiamond type—H_2, concentration—167 ng/μL, incubation time—3 h.

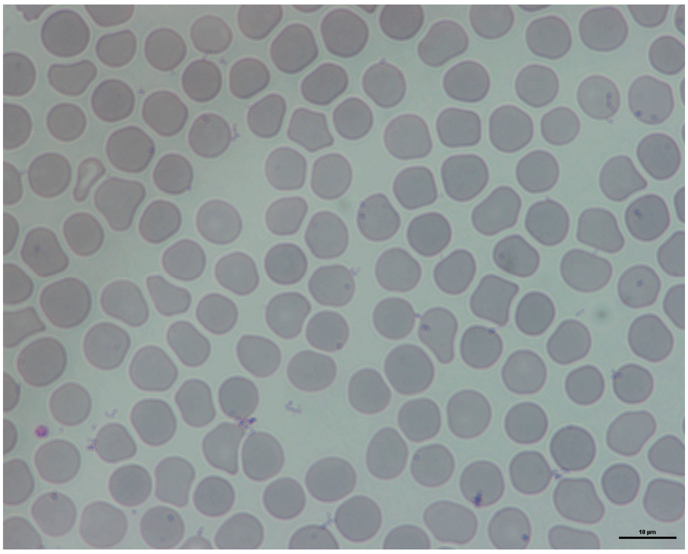

Figure S31. Nanodiamond type—H_2, concentration—167 ng/μL, incubation time—5 h.
